# Practical considerations for plant phylogenomics

**DOI:** 10.1002/aps3.1038

**Published:** 2018-04-02

**Authors:** Michael R. McKain, Matthew G. Johnson, Simon Uribe‐Convers, Deren Eaton, Ya Yang

**Affiliations:** ^1^ Department of Biological Sciences The University of Alabama Box 870344 Tuscaloosa Alabama 35487 USA; ^2^ Department of Biological Sciences Texas Tech University 2901 Main Street, Box 43131 Lubbock Texas 79409 USA; ^3^ Department of Ecology and Evolutionary Biology University of Michigan 830 North University Ann Arbor Michigan 48109 USA; ^4^ Department of Ecology, Evolution, and Environmental Biology Columbia University 1200 Amsterdam Avenue New York New York 10027 USA; ^5^ Department of Plant and Microbial Biology University of Minnesota–Twin Cities 1445 Gortner Avenue St. Paul Minnesota 55108 USA

**Keywords:** genome skimming, microfluidics, phylogenomics, RAD‐seq, sequence capture, transcriptomes

## Abstract

The past decade has seen a major breakthrough in our ability to easily and inexpensively sequence genome‐scale data from diverse lineages. The development of high‐throughput sequencing and long‐read technologies has ushered in the era of phylogenomics, where hundreds to thousands of nuclear genes and whole organellar genomes are routinely used to reconstruct evolutionary relationships. As a result, understanding which options are best suited for a particular set of questions can be difficult, especially for those just starting in the field. Here, we review the most recent advances in plant phylogenomic methods and make recommendations for project‐dependent best practices and considerations. We focus on the costs and benefits of different approaches in regard to the information they provide researchers and the questions they can address. We also highlight unique challenges and opportunities in plant systems, such as polyploidy, reticulate evolution, and the use of herbarium materials, identifying optimal methodologies for each. Finally, we draw attention to lingering challenges in the field of plant phylogenomics, such as reusability of data sets, and look at some up‐and‐coming technologies that may help propel the field even further.

Phylogenomics, or using genome‐scale sequence data for phylogenetic analyses, has seen major advancements in recent years. Because of the rapid improvement of high‐throughput sequencing (HTS) platforms, reduced representation strategies, and analytical tools, obtaining hundreds to thousands of loci has become routine for many botanical researchers. As of early 2018, Illumina HiSeq and MiSeq short‐read technologies (Illumina Inc., San Diego, California, USA) are the workhorses of phylogenomics. Emerging long‐read technologies such as Pacific Biosciences (Menlo Park, California, USA; PacBio hereafter) and Oxford Nanopore Technologies (Oxford, United Kingdom; Nanopore hereafter) are facilitating acquisition of long loci as well as improved assembly of whole genomes. A number of analytical approaches have been developed to detect polyploidy and dissect heterogeneity within phylogenetic data sets (Li et al., 2015; Smith et al., 2015; McKain., 2016b; Gompert and Mock, 2017; Gregg et al., 2017), making it easier to address polyploidy and reticulate evolution using genome‐scale data. Additionally, the community is pushing for full open access to both data and computer code, making it timely to discuss the tradeoffs each strategy has in terms of resolving complicated evolutionary history and reusability of data. With rapidly evolving new tools and the caveats that they bring, choosing an optimal strategy that takes into consideration cost, available plant tissue, and short‐ and long‐term research goals can be a daunting task, especially for people who are new to the field of phylogenomics.

In this review article, we focus on the costs and benefits of different approaches used in phylogenomics including: microfluidic PCR, restriction enzyme–based methods, genome skimming, target enrichment, and transcriptomics. We highlight unique challenges and opportunities in plant systems—such as polyploidy, which requires distinguishing homeologous gene copies; reticulate evolution, which requires biparentally inherited loci; and the use of herbarium materials with short and partially degraded DNA—making practical suggestions for each. Finally, we draw attention to challenges such as reusability of data sets and discuss some up‐and‐coming technologies that may help propel the field even further. Table 1 provides a side‐by‐side comparison of the methods explored in this manuscript.

**Table 1:** Comparison of cost and utility of phylogenomic methods for plants.^a^

Aspect of method	Sanger‐based methods	Microfluidic PCR	Restriction enzyme–based methods	Genome skimming	Target enrichment	Transcriptome
Upfront investment	Design and optimizing PCR primers. Timeframe: weeks; cost: $50–100	Some genomic data (e.g., shotgun libraries). Timeframe: weeks to months; cost: $100–1000	Optional: test alternative restriction digestions for optimal range of fragment sizes. Timeframe: weeks; cost: $100–1000	None	Transcriptome and/or genomes from closely related organisms. Timeframe: months; cost: $100–1000	Freezers and liquid nitrogen containers; logistics for tissue collecting. Timeframe: weeks to months; cost: $1000–10,000
Tissue for sampling: herbarium, silica preserved, flash‐frozen, living	All four types but reduced success from low‐yield herbarium tissue extractions	All four types, but reduced success from low‐yield herbarium tissue extractions	All four types, but reduced success from low‐yield herbarium tissue extractions	All four types, but potential reduced success from low‐yield herbarium tissue extractions. See Saeidi et al., 2018.	All four types, but reduced success from low‐yield herbarium tissue extractions	Flash‐frozen or living tissue preserved in RNA*later*
Sequence information type	Coding region, short introns, and short intergenic spacers	Coding region, introns, and short intergenic spacers	Anonymous or reference‐mapped short‐length loci	Organellar, some nuclear	Coding region and flanking intron	Coding region
Cost per extraction + library prep + sequencing (varies depending on platform)	$1–5 for standard DNA extraction + $0 + $3 for a single read of 800–1000 bp	$1–5 for standard DNA extraction + $0 + $0.40 per microfluidic reaction (~$800 per 48 × 48 plate)	$1–5 for standard DNA extraction + $5–50 + $1200–1800 (HiSeq sequencing 48–384 samples)	$1–5 for standard DNA extraction + $25–150 + $50 (assume 1.5–2 Gb of data per sample)	For 96 samples: $200 for probes, $1–5 for standard DNA extraction + $16 (library in 1/3 volumes) + $1800 for sequencing (MiSeq 2 × 300)	$5–15 for RNA extraction + $50–150 + $200 (assume 25 million reads per transcriptome)
Assembly and cleaning data	Easy	Easy	Relatively easy	Moderately computationally intensive	Moderately computationally intensive	Computationally intensive
Ability to resolve reticulation/hybridization/introgression	Yes	Yes, potential to recover single alleles from nuclear loci	Yes, significant power to test for genome‐wide or localize admixture	Sometimes, potentially identify hybridization but only if it is biparentally inherited	Yes, can extract alleles if long reads are used	Yes
Ability to infer polyploidy	Yes, but needs time‐consuming cloning	Yes, potential to recover single alleles from nuclear loci	Sometimes, can detect polyploidy from read depths, but low potential to separate paralogs	No	Sometimes, can detect abundance of paralogous sequences	Sometimes, if polyploidy event is old enough that homeologs can be separated during transcriptome assembly
Best use	First pass; when maximizing the number of samples is the priority	Closely related species; studies that need specific loci and complete data matrices	Shallow phylogenetic scale with aim to sample many individuals	Deep or shallow phylogenetic scale; detecting parental heritage; potential genome diversity	Deep or shallow phylogenetic scale for up to a few samples per species	When detecting genome duplication and gene family evolution are of interest beyond reconstructing species relationship
Reusability of data	Yes, if using the same loci	Yes, fully reusable within focus group using same loci, limitations with increase in phylogenetic distance	Sometimes, reusable for studies within same study system, not reusable between distant clades	Yes, fully reusable	Sometimes, partially reusable across studies if same loci are targeted	Yes, fully reusable

a Costs are given in U.S. dollars (US$) as of 2018.

## SANGER SEQUENCING

Primer design from available data → DNA extraction, PCR amplification, and sequencing → read consolidation → phylogenetic inference

### In many cases a few loci are sufficient

Low‐throughput sequencing‐based approaches, typically by PCR amplification of nuclear ribosomal ITS (Baldwin et al., 1995; Baldwin, 1998) and/or a handful of chloroplast regions (Shaw et al., 2005, 2007, 2014) followed by Sanger sequencing, are still commonplace in plant systematics. These methods have the advantages of being low cost (despite relatively high cost per base compared to HTS), requiring only standard molecular lab equipment, and having straightforward data analysis. These methods are often used as the first step in molecular systematics training for students and are useful in many cases for phylogenetic reconstruction. In addition, Sanger‐based methods can be used for barcoding vegetative or fragmented samples, or as a quick first pass for selecting samples for collecting genome‐scale data.

Due to frequent polyploidy and reticulate evolution in plants, amplified nuclear regions often contain co‐amplified paralogs and divergent alleles that must be isolated by laborious procedures like cloning. For this reason, PCR amplification of low‐copy genes—a common practice in animal phylogenetics—is not particularly efficient. To avoid cloning, high‐throughput single‐molecule sequencing approaches, like PacBio, have been effective in sequencing and isolating homeologs from amplified PCR products for a small number of loci in polyploid taxa (Rothfels et al., 2017). For many phylogenetic analyses, however, a lack of phylogenetic signal and potentially high conflict found among few nuclear and chloroplast regions necessitates sequencing a larger number of loci, i.e., a phylogenomics approach. Medium‐throughput approaches can be employed by performing PCR amplification of multiple loci per taxon and barcoding them for pooling and sequencing on platforms like the Illumina MiSeq (e.g., Cruaud et al., 2017). However, as overall preparation time and costs for more high‐throughput methods continue to decrease, HTS becomes a more cost‐effective choice.

## MICROFLUIDIC PCR

Primer design from available data → DNA extraction, PCR amplification, and sequencing → read/locus alignment → phylogenetic inference

Among the multiple phylogenomic approaches commonly used today, microfluidic PCR is one that has a familiar feel to it. As the name implies, it is based on PCR amplification of targeted regions but has the advantage of producing much larger amounts of data more efficiently and cost‐effectively than standard PCR. Microfluidic PCR is based on the same principles as standard PCR: a DNA template, a set of forward and reverse primers used to target and amplify the region of interest, enzymatic and chemical reagents (i.e., *Taq* polymerase and dNTPs), and a series of heating and cooling steps. The main differences between the two are that (1) two pairs of primers (two forward and two reverse) are used in each microfluidic reaction instead of just one, (2) the volumes of DNA template and other reagents are extremely reduced, (3) all primer pairs have the same annealing temperature, and (4) amplified regions should be of similar lengths. Microfluidic technology has become popular in biomedical fields like cancer research (e.g., Gaedcke et al., 2012; Walter et al., 2012), genotyping of single‐nucleotide polymorphisms (SNPs) (e.g., Bhat et al., 2012; Byers et al., 2012; Lu et al., 2012), gene expression (e.g., Dominguez et al., 2013; Moignard et al., 2013; Shalek et al., 2013), and targeted resequencing (e.g., Lohr et al., 2012; Moonsamy et al., 2013). Recently, this technology has been used for generating phylogenetic data sets in microbial systems (Hermann‐Bank et al., 2013), haplotyping (identification of individual genome copies) of commercially important plants (Curk et al., 2014), and elucidating recent radiations in plants (Gostel et al., 2015; Uribe‐Convers et al., 2016).

### Capacity of microfluidic PCR

In phylogenomics, the most commonly used equipment for microfluidics is the Fluidigm Access Array System (Fluidigm Corporation, San Francisco, California, USA). This machine functions as a standard thermocycler except that it amplifies 48 target regions across 48 accessions/taxa (2304 amplicons) per microfluidic array, whereas other versions of this equipment may have higher throughput (e.g., 96 × 96). Each reaction is performed with four primers simultaneously: a pair of primers that amplify the region of interest and a second pair of primers that anneal to the first pair adding barcodes and sequencing adapters to the amplicon. Because the final product contains barcodes, sequencing adapters, and the region of interest, this four‐primer PCR approach circumvents the need for other library construction methods. After PCR amplification, all amplicons from each sample are combined in a single pool, and all 48 pools of amplicons (one for each sample) are quantified and sequenced on an HTS platform. The current yield of HTS platforms (e.g., Illumina MiSeq) allows for up to four to six microfluidic plates to be sequenced on one lane and still get the necessary sequencing depth for phylogenomics. Lastly, amplification reactions on the Fluidigm Access Array System can be multiplexed. Fluidigm has shown that one could combine up to 10 primer pairs per well—bringing up the number of amplicons to 23,040 per microfluidic plate—if the primers within a well have no interaction (e.g., primer dimers) and if the target regions are located far enough in the genome so that PCR amplifications do not interfere with each other.

One of the main advantages of microfluidic PCR is that minimal quantities of DNA and PCR reagents are used. The 2304 wells available in the Fluidigm Access Array 48 × 48 plate are only 36 nL in total volume (Cronn et al., 2012), and require just 15 units of *Taq* polymerase to amplify the entire plate. Similarly, only 1 μL of DNA template is necessary to generate all amplicons for each sample. Although Fluidigm recommends that high‐quality (50 ng/μL) DNA be used in the reactions, there has been success with much lower concentrations (~10 ng/μL), and importantly, with DNA extracted from herbarium specimens (Uribe‐Convers et al., 2016; Latvis et al., 2017).

### Microfluidic PCR produces consistent data sets across lineages

Data produced by microfluidic PCR are ideal for phylogenomics. Amplified and sequenced regions have been through a rigorous selection process, often targeting single‐ or low‐copy loci that are highly informative, resulting in data that are useful to resolve relationships in both young and old clades. Moreover, only targeted regions are amplified and sequenced, increasing both the sequencing depth for each amplicon and the number of loci that are shared among samples. This last point is particularly important as it reduces the amount of missing data in alignment matrices—something that has been shown to be beneficial for phylogenetic inference (Lemmon et al., 2009), although not always a necessity for topology reconstruction (Rubin et al., 2012; Mastretta‐Yanes et al., 2015).

Another key advantage of microfluidic PCR is that it can be used with polyploid species. As mentioned above, the highly targeted nature of this approach yields consistently deep sequencing depth among all samples, which facilitates recovery and identification of alleles and homeologs within a locus. Sequence data from microfluidic PCR can be demultiplexed twice: first using the sequencing barcodes and then using the PCR primer sites. This double demultiplexing approach results in groups of sequencing reads that belong to a specific sample and locus, reducing complexity for assembly. If the HTS is done with relatively long (300‐bp reads on MiSeq) paired‐end reads, no read assembly is needed, except for potentially collapsing reads. By simply aligning the reads back to a reference, many of the computational burdens that an assembly entails are greatly minimized. Variant call information for a locus within a sample can be used to identify different alleles. This approach has been demonstrated using the young genus *Neobartsia* Uribe‐Convers & Tank (Orobanchaceae) with great success, and scripts are available to process microfluidic data (Uribe‐Convers et al., 2016). Additionally, microfluidic PCR has been used to study the evolutionary histories of *Commiphora* Jacq. (Burseraceae; Gostel et al., 2015) and squashes (*Cucurbita* L., Cucurbitaceae; Kates et al., 2017). Finally, once a set of primers are available, they can be re‐used within the same group and, in some cases, among allied genera within a family (Latvis et al., 2017) and even an order (Collins et al., 2016).

### Upfront investment in primer design

The main disadvantage of microfluidic PCR compared to other HTS methods is the time invested in primer design. Compared to standard PCR primers, all microfluidic primer pairs must have an annealing temperature of 60°C (±1°C). Higher annealing temperatures usually require designing longer primer sequences (~27 bp) in conserved genomic regions, which can be difficult to find across taxa. To compound the challenges of primer design, the regions that are targeted for amplification should all be close in length (usually ~600–900 bp). The latter point is important because sequencing depth could be biased toward shorter regions (~350 bp), or if the regions are too long (>1000 bp), they could interfere with each other during bridge amplification on an Illumina platform. Some prior sequence data (e.g., plastomes, shotgun libraries, transcriptomes) from three to five species within a focal clade are required for designing primers in order to maximize amplification success across a clade. It is recommended to validate primer amplification by simulating the microfluidic PCR conditions in a standard thermocycler. A good primer pair should not create primer dimers nor interfere with barcode and sequencing adapter primers. A good primer pair should also only amplify the region of interest (i.e., no double bands on an agarose gel). Finally, microfluidic amplification is done in a specialized piece of equipment (e.g., Fluidigm Access Array System) that might not be available in standard molecular laboratories or genomic cores. However, microfluidic instruments are used in other amplification‐based studies (e.g., genotyping or variant calling), and they are becoming more popular and even commonplace in large genomic cores. Although a researcher new to microfluidics might at first feel discouraged by its limitations, this method provides data matrices with very little missing data, allows complete control over what loci get sequenced, and has no assembling steps and minimal data processing, and the resulting data can outweigh the initial difficulties of primer design.

## RESTRICTION ENZYME–BASED METHODS

Restriction enzyme selection→ DNA extraction→ DNA digestion → library preparation and sequencing → read mapping/SNP identification → phylogenetic inference

High‐throughput restriction‐site‐associated DNA sequencing describes a suite of related methods, here referred to collectively as RAD‐seq, that utilize restriction enzymes to fragment genomic DNA prior to sequencing (Miller et al., 2007; Baird et al., 2008; Davey et al., 2011; Andrews et al., 2016). Illumina adapters are ligated to digested fragments during library preparation such that all sequenced reads begin at restriction cut sites and extend away for the length of a single sequenced read (typically 75–300 bp). The resulting sequenced reads therefore accumulate at each RAD locus to form high‐coverage stacks that can be used to confidently call alleles and SNPs—a convenient outcome relative to many other genomic methods that require tiling partially overlapping sequences, often at lower depths, to construct contigs. Due to their relatively short lengths, however, RAD loci are often not highly informative for constructing gene trees, and instead are typically best suited for SNP‐based inference methods (discussed further below). Nevertheless, because RAD‐seq methods are affordable and easy to implement, they have been widely adopted in population genetics, phylogeography, and phylogenetics (Hohenlohe et al., 2010; Eaton and Ree, 2013; Nadeau et al., 2013; Wagner et al., 2013; Hipp et al., 2014; Herrera et al., 2015; Leaché et al., 2015; McCluskey and Postlethwait, 2015; DaCosta and Sorenson, 2016).

### High flexibility and low cost

One of the greatest strengths of RAD‐seq is its flexibility. If you wish to sample more genetic markers, you can simply select a more frequently cutting restriction enzyme or a wider window of digested fragment sizes to include in the sequenced library. If instead you want fewer markers sequenced to higher depth, you can choose a less common cutter. In this way, for most large plant genomes, it is possible to target approximately as many RAD loci for inclusion in your data set as you see fit. This has made RAD‐seq particularly promising for the study of recent radiations (Nadeau et al., 2013; Wagner et al., 2013), species delimitation (Leaché et al., 2014), and introgression/admixture (Dasmahapatra et al., 2012; Eaton and Ree, 2013), where a broad sampling of sites from thousands of regions across the genome can be used to characterize heterogeneity in the distribution of gene tree patterns and provide statistical power for tests of reticulate evolution (Durand et al., 2011; Reddy et al., 2017).

The flexibility of RAD‐seq can sometimes also lead to confusion as there are now a variety of related methods with similar names but distinct differences. These protocols typically vary in the number and type of enzymes that are used, as well as in the equipment that is required for their preparation, all of which can lead to significant differences in their cost as well as in the quality and quantity of data generated (Elshire et al., 2011; Peterson et al., 2012; Toonen et al., 2013; Andrews et al., 2016; Glenn et al., 2017; see Andrews et al. 2016 for a review). For example, the original RAD protocol uses only a single restriction enzyme to produce a range of DNA fragment sizes that are subsequently subjected to sonication in order to shear DNA fragments to the appropriate length for short‐read sequencing. In contrast, dual‐digest methods use two restriction enzymes to digest the genome into variably sized fragments. The resulting fragments are selected based on whether their size falls within an appropriate window for short‐read sequencing. The former method can provide more data with less bias caused by mutations to restriction sites, while the latter method can be more flexible in tailoring the number of selected fragments and is cheaper because it requires less specialized equipment and adapters.

The relatively low cost of RAD‐seq is one of the primary reasons it has attracted significant use in recent years. The initial cost of materials is low. Subsequent library preparations and sequencing typically range from US$15–75 per sample, and the cost continues to decrease (Andrews et al., 2016). Recent advances to indexed primers (Glenn et al., 2016) and library preparations (e.g., 3RAD; Glenn et al., 2017) have further decreased costs to below US$1/sample for large multiplexed data sets (Hoffberg et al., 2016b). A potentially promising approach for large‐scale projects, in which many hundreds or even thousands of individuals are to be sequenced, is to use a hybrid approach like RADcap, which combines restriction digestion with targeted bait capture (Hoffberg et al., 2016a). This combined approach to select and sequence fewer RAD loci provides more even sequencing coverage and allows more samples to be multiplexed. Depending on the number of samples in a study and the number of loci to be targeted, selecting an appropriate protocol can be hugely beneficial and cost saving.

### Analysis of RAD‐seq data

A major strength of RAD‐seq is the enormous quantity of data that can be collected. Because the method does not rely on targeting relatively invariant regions of the genome or coding genes, there are often high rates of variation across RAD loci that make it easy to sample thousands or even millions of SNPs. Typical assembly methods for RAD‐seq provide both SNP‐based sequence formats and full sequence data for traditional phylogenetic analyses (Catchen et al., 2013; Eaton, 2014), with the addition that reference‐mapped data provide the genomic location of markers. In this way, the distribution of sampled RAD loci and SNPs across chromosomes can also be used to study phylogenetic relationships or patterns of admixture as they vary across sliding windows of the genome (e.g., Dasmahapatra et al., 2012). Large SNP data sets may prove particularly useful as SNP‐based phylogenetic inference methods continue to develop (see review by Leaché and Oaks, 2017). Such methods that do not require inferring fully resolved gene trees for each locus can expand the utility of methods like RAD‐seq while also reducing errors in phylogenetic analyses that arise from assuming gene trees are accurate and that recombination is absent within longer sequences of DNA (Bryant et al., 2012; Chifman and Kubatko, 2014). Still, for relatively deeper‐scale phylogenetic analyses, RAD‐seq loci are often sufficiently variable to be used in gene‐tree‐based methods that employ the multi‐species coalescent (e.g., Ogilvie et al., 2016; Eaton et al., 2017; Vargas et al., 2017).

### Missing data require careful filtering

A particularly relevant concern for RAD‐seq is missing data, although the implications of missing data apply similarly to any phylogenomic analysis. Because RAD‐seq relies on the conservation of restriction recognition sites across samples in order to target homologous markers, disruption of these sites by mutations (mutation‐disruption) leads to missing data. The generation of new restriction recognition sites by mutations can also lead to RAD loci being shared by some samples and not others, but simulations suggest that disruption of ancestrally shared restriction sites is of much greater significance (Eaton et al., 2017). More divergent taxa are thus expected to share fewer conserved restriction sites on average, and thus less pairwise phylogenetic information. This has led to considerable debate as to whether RAD‐seq can be accurately applied to deeper phylogenetic scales (Rubin et al., 2012; Cariou et al., 2013). Although it is now clear from empirical applications that RAD‐seq can provide significant phylogenetic information over even tens of millions of years divergence (Eaton and Ree, 2013; Escudero et al., 2014; Eaton et al., 2015, 2017; McVay et al., 2017; Tripp et al., 2017; Vargas et al., 2017), the more relevant concern is the scale at which missing data make this method no longer economical compared to alternatives.

Although the problem of missing data is inherent to RAD‐seq data sets, in most cases many thousands of loci can be recovered across all or nearly all samples in a study. Bioinformatic methods are employed to filter loci from a data set to select those with some minimum proportion of missing data (Eaton, 2014). Depending on how many restriction enzyme cut sites are targeted, the quality of the library, the sequencing coverage, and the number of samples and their phylogenetic relationships, this may constitute a large proportion of the total loci or a very small proportion (sometimes even none). However, because loci with missing information for some taxa can still provide phylogenetic information for many other taxa, most data sets allow for 30–90% missing data in combined multi‐locus data sets (Eaton et al., 2017). In general, missing data tend to have little impact on phylogenetic tree topology (Rubin et al., 2012; Mastretta‐Yanes et al., 2015) but can significantly affect other aspects of phylogenetic inference such as branch lengths (Ogilvie et al., 2016).

Although the primary source of missing data in RAD‐seq studies is typically assumed to be mutation‐disruption, many other aspects of library preparation or sequencing can have an equal or greater effect. Consider that one of the drawbacks of RAD‐seq is that loci contain very little variation—only one or a few SNPs per locus. This presents a contradiction to the expectation that mutation‐disruption causes most missing data: if few mutations occur in a 100‐bp locus, then even fewer mutations should occur in the small restriction recognition site adjacent to the locus. Thus, mutation‐disruption would be unlikely to cause 50% of sequences to be missing from a data set. Instead, the amount of missing data in RAD‐seq will often depend on many other factors.

In a comparison of 10 phylogenetic‐scale RAD‐seq data sets, Eaton et al. (2017) showed that selecting an appropriate library preparation method and sequencing depth is of great significance for the amount of phylogenetic information that will be obtained. For example, in a relatively deep‐scale phylogenetic analysis of the genus *Viburnum* L., they showed that a 2× increase in sequencing coverage led to >10× increase in the number of phylogenetically informative sites recovered. However, the same return on sequencing coverage was not observed for all data sets, with the primary difference depending on whether the library was single or dual digest. Single‐digest libraries tend to generate many fragments that are often under‐sequenced, whereas dual‐digest libraries usually select many fewer fragments that are more easily sequenced to sufficient depth. Although the number of reads that must be sequenced to attain a sufficient level of coverage per sample can be estimated based on the expected number of loci in a genome, such estimates are often difficult to make, and it is typically easier to simply base estimates on studies already conducted in related organisms. A discussion of differences among RAD‐seq protocols is clearly relevant for designing a project, as well as when comparing RAD‐seq to other methods. Although RAD‐seq methods are easy to implement, careful attention and troubleshooting of library preparations, including the quality of DNA extractions, restriction digestions, and size selection windows, can have an enormous effect on the results (Graham et al., 2015).

### Working with duplications and paralogy

Due to the frequency of gene and genome duplications in plants, anonymous phylogenetic markers have historically received relatively little use, and for many researchers, this reticence applies similarly to RAD‐seq. However, anonymity is not necessarily a property of RAD‐seq per se, but rather a possible outcome depending on how the data are assembled. If a good reference genome is available, RAD loci can be assembled like many other genomic markers by mapping reads to a reference, in which case paralogy is assessed based on whether reads map to multiple locations in the genome. Due to their short length, however, paralogous loci are typically removed from RAD‐seq data sets rather than being further analyzed to try to tease apart paralogous gene tree histories. It is only for cases in which taxa lack a good reference genome that RAD loci are assembled de novo, wherein reads are clustered by sequence similarity to identify homology. Paralogy is more difficult to assess in this case and is usually based on distributions of site frequencies and excesses of heterozygosity or alleles (Eaton, 2014).

Empirically, the effect of paralogs on phylogenetic inference is difficult to assess, as there are many possible ways in which paralogs can be distributed. In their recent phylogenetic study of the plant clade *Viburnum*, Eaton et al. (2017) compared a RAD‐seq phylogeny to a tree inferred from Sanger sequence data composed of a nuclear locus and chloroplast sequences (ITS + nine cpDNA regions) and found nearly complete concordance, despite the fact that *Viburnum* has several instances of derived polyploidy. From this, they suggested that any paralogs retained in the RAD data set after filtering likely had relatively little effect on the genome‐wide phylogenetic signal. The ratio of phylogenetic signal to noise generated by ancient genome duplications versus more recent species divergences will typically determine the extent to which paralogy is likely to obscure phylogenetic inference. It remains for more detailed studies to investigate the impact of paralogy on various phylogenomic data sets analyzed under different methods. Most applications of RAD‐seq for polyploids to date have focused on the detection of ploidy based on read‐depth information (Gompert and Mock, 2017); however, there remains a lack of phylogenetic methods for further analysis of polyploids using primarily SNP data.

## GENOME SKIMMING

DNA extraction, library preparation, and sequencing → organelle genome assembly → annotation → phylogenetic inference

Genome skimming (also called genome survey sequencing or low‐coverage genome shotgun sequencing) is the method of sequencing total genomic DNA without any enrichment (Straub et al., 2012). In plants, the resulting data are a representation of the nuclear, chloroplast, and mitochondrial genomes of the target individual, although contaminants from pathogens, the microbiome, and symbionts (e.g., Nakamura et al., 2013) may also be present. Often, genome skim data contain less than 1× coverage of the nuclear genome, making them inadequate for identification of nuclear genes. However, when sequenced at a higher depth (2–3×), Berger et al. (2017) have demonstrated that it is possible to extract low‐copy nuclear genes. Higher coverage is needed not only to ensure complete representation of low‐copy loci but also to overcome issues of sequencing error. Other fractions of the data, such as the chloroplast genome (McKain et al., 2016a; Qu et al., 2017), mitochondrial genome (Guo et al., 2016; Petersen et al., 2017), nuclear ribosomal genes (Steele et al., 2012), and repetitive elements (e.g., transposable elements), are in much higher copy numbers, are generally represented in higher relative coverage compared to low‐copy loci (>30×), and often allow for assembly of organellar genomes and ribosomal genes or characterization of transposon diversity and quantity (Staton and Burke, 2015b). Genome skimming is a relatively easy first step into phylogenomics because projects require commonly used molecular techniques such as DNA isolation and sequencing library production and do not require data generation prior to initiating a project.

### Multiple types of specimens are viable

A benefit of genome skimming is that any source of viable double‐stranded DNA can be used. Projects using living (Male et al., 2014; Zhang et al., 2015) and herbarium (Bakker et al., 2016; Teisher et al., 2017) specimens have all successfully generated genome skim data (Staats et al., 2013). Genome skimming is able to use DNA that is otherwise too degraded for PCR‐based sequencing approaches (Staats et al., 2011). In recent years, the use of herbaria in genome skim projects has exploded, with molecular workflows modified for isolation and shotgun sequencing of herbarium DNA (Särkinen et al., 2012; Bakker et al., 2016; Saeidi et al., 2018) producing whole chloroplast genomes from samples more than 100 years old (Bakker et al., 2016), endangered species (Welch et al., 2016), and extinct species known only from herbarium collections (Zedane et al., 2016). Because these workflows produce viable libraries for HTS, they are also amenable to sequence capture, as discussed below.

### Chloroplast genomes are readily and inexpensively obtained

Organellar genomes make up a large component of total genomic DNA, with cpDNA ranging from <0.3% in *Picea abies* (L.) H. Karst. needles to 37% in *Asclepias syriaca* L. leaves (Twyford and Ness, 2016) and mitochondrial DNA abundance 5–10% that of cpDNA (Bock et al., 2014). Although genome size in flowering plants varies from 63.6 Mbp in *Genlisea aurea* A. St.‐Hil. (Leushkin et al., 2013) to almost 152.23 Gbp in *Paris japonica* (Franch. & Sav.) Franch. (Pellicer et al., 2010), there does not appear to be a direct negative correlation between genome size and percent total organellar DNA, suggesting that genome skimming is a viable option for obtaining organellar genomes across many taxa (Bakker et al., 2016; Twyford and Ness, 2016). Because of their relative abundance, (usual) structural simplicity, and historical significance in systematics, chloroplast genomes have become a primary target of genome skimming projects.

When designing projects for chloroplast genome sequencing, two factors should be considered to maximize data over cost. First and foremost, it must be decided if full chloroplast genomes are necessary or if protein‐coding gene space will be adequate. Acquiring full chloroplast genomes for each sample can add extra time to data generation and analysis; however, multiple assembly pipelines (e.g., ACRE [Wysocki et al., 2014], IOGA [Bakker et al., 2016], NOVOPlasty [Dierckxsens et al., 2017], Fast‐Plast [https://github.com/mrmckain/Fast-Plast], and a *k*‐mer‐based approach [Izan et al., 2017]) have been developed that are capable of assembling complete chloroplast genomes from short‐read data. Complete chloroplast genomes can potentially provide more phylogenetic signal from intergenic regions for reconstructing relationships among closely related species (Carbonell‐Caballero et al., 2015). When sequencing of complete chloroplast genomes is not feasible or necessary, read mapping–based approaches can provide adequate assemblies for chloroplast gene space for population‐level studies (Vallejo‐Marín et al., 2016). De novo assemblies of complete chloroplast genomes often require higher coverage than mapping‐based approaches, so this must be taken into account in project design. The second factor to consider is the relative percentage of total genomic DNA that is chloroplast for the taxa being sequenced. An underestimate can result in chloroplast genomes not sequenced deeply enough for adequate data acquisition, and an overestimate can result in wasted potential taxon sampling. In practice, whole chloroplast genomes can be assembled from an estimated 50–100× average coverage, although coverage is not always consistent across the chloroplast genome. Regions rich in either AT or GC repeats often see decreases in coverage (Benjamini and Speed, 2012) meaning some lineages may need a higher average coverage for complete chloroplast genome assembly. Estimating the percentage of total DNA can be done by mapping existing reads from the taxon group to a representative chloroplast genome (e.g., Twyford and Ness, 2016). If such data are not available, quantitative PCR (qPCR) can be used to estimate the relative percentage of chloroplast DNA in a sample (Lutz et al., 2011). Through qPCR, cpDNA percentage for each taxon can be estimated, allowing for optimal multiplexing of samples to relatively equal chloroplast read representation across taxa. Ultimately, the number of taxa in a sequencing run is related to the sequencing potential of the run (i.e., total reads and read length), the average size of the chloroplast genome for the group, the desired average coverage of the chloroplast genome, and the relative percentage of total genomic DNA that comes from the chloroplast. An Illumina run of 100 million 150‐bp paired‐end reads is capable of sequencing 50–60 samples for complete chloroplast genomes (Teisher et al., 2017), although this will vary among taxa and DNA source (fresh vs. herbarium).

### More than just chloroplast genomes

In addition to chloroplast genomes, genome skimming provides a first look at genome composition. Genome skimming provides data for both development of target enrichment probes (Schmickl et al., 2016) and the isolation of low‐copy nuclear genes given sufficient coverage (Berger et al., 2017). Transposable element diversity and composition can also be discerned through genome skim data. Although multiple studies have focused on the use of longer read technology such as 454 pyrosequencing (e.g., Harkess et al., 2016), the development of RepeatExplorer (Novák et al., 2013) and Transposome (Staton and Burke, 2015b) enable short reads to be used for transposon identification (e.g., Staton and Burke, 2015a). Using these approaches, genome skim data are able to provide novel insights into the evolution of nuclear genomes, not just organellar genomes. When used in conjunction with chloroplast‐based phylogenies derived from the same data, understanding of transposon and genome evolution is greatly extended. These types of analyses are specifically suited to genome skimming, which provides unbiased genome sampling compared to enrichment‐based approaches. Genome skimming also allows for the assembly of other highly repetitive nuclear regions, such as nuclear ribosomal DNA (Kim et al., 2015), potentially providing phylogenetically informative nuclear loci.

### Limitations caused by non‐biparental inheritance in reconstructed phylogenies

As in most sequencing‐based projects, some taxa can be difficult to work with and not all samples will result in useful data due to DNA quality. Genome skimming can provide complete chloroplast genome sequences, but these can be limiting in the resolution they offer to phylogenomics projects if hybridization and polyploidy are abundant. Chloroplast genomes are usually uniparentally inherited, which becomes problematic in the identification of hybridization and polyploid events. Phylogenetic signal from whole chloroplast genomes can, at times, suggest incomplete lineage sorting or introgression (i.e., chloroplast capture), especially at the inter‐ and intraspecific levels (Wambugu et al., 2015; Zhou et al., 2017). It can be difficult to tease apart incomplete lineage sorting and hybridization because they display similar phylogenetic patterns, especially if only chloroplast genomes are considered. In cases in which bidirectional hybridization occurs and both parental species donate chloroplast genomes, it may be possible to detect a hybridization event using chloroplast‐based phylogenies. However, a combined approach of nuclear and chloroplast genomes will be much more powerful than either alone. As such, both the biology of the species (i.e., hybridization frequency, recent radiations) and the research questions must be amenable to organellar‐based phylogenies for genome skimming to be successful. It should be noted, however, that chloroplast phylogenies often recover comparable phylogenetic relationships to those seen in nuclear‐based studies (e.g., Gitzendanner et al., 2018), suggesting that these limitations are situation specific. Although the use of genome skimming for transposon diversity studies is promising, the methodology has primarily been tested in lineages with well‐documented transposons (e.g., Asteraceae and Poaceae) and may be less accurate in understudied lineages due to fewer or no reference genomes being available.

## TARGET ENRICHMENT

Probe design from available data → DNA extraction, library preparation, hybridization, and sequencing → locus assembly → homology and orthology inference → phylogenetic inference

Although genome‐skimming methods are useful for extracting phylogenetic data from organellar and other high‐copy regions in plants (Stull et al., 2013), they are not typically feasible for recovering nuclear genes. Several methods have emerged for enrichment of shotgun (i.e., Illumina) sequencing libraries for genes of interest, including ultraconserved elements (Faircloth et al., 2012), anchored phylogenomics (Lemmon et al., 2012), exon capture (Mandel et al., 2014), and Hyb‐Seq (Weitemier et al., 2014). Each of these methods, which we will collectively refer to as “target enrichment” (Mamanova et al., 2010), work via the use of short (60–120 bp) RNA probes that hybridize to sequence library fragments. The hybridized fragments are typically bound to magnetic beads while the remainder of the library is discarded. Commonly used target enrichment methods differ in the types of DNA that are targeted. Ultraconserved elements (UCEs) and anchored phylogenomics target slow‐evolving genomic regions (that may or may not be associated with protein‐coding genes) using universal probes that can be used across a wide phylogenetic diversity of organisms. These methods rely on sequence variation in regions flanking the conserved genomic elements. In contrast, protein‐coding genes are used to design probes for exon capture and Hyb‐Seq approaches. Depending on the phylogenetic breadth of the study, the exon data may be used directly. Alternatively, flanking intron regions are also captured and can be useful for informing more recent relationships. In the Hyb‐Seq approach, exon capture is combined with analysis of off‐target organellar reads to retrieve both nuclear and organellar data in the same sequencing run.

### Consistently recovering large regions from multiple DNA sources

As with PCR‐based methods, target enrichment requires some prior genomic knowledge about the target organisms. Although several genomes spanning the breadth of target organisms are required for probe design for the UCE and anchored phylogenomics methods, they are not required for Hyb‐Seq. Furthermore, it has proven difficult to identify ultraconserved elements in plants, likely due to the high amounts of genome duplication. For target enrichment in plants, many groups have instead chosen to focus on low‐copy protein‐coding genes, using exon capture or Hyb‐Seq designs (Johnson et al., 2016; Crowl et al., 2017; Landis et al., 2017; Villaverde et al., unpublished manuscript). Target enrichment has also been used to capture chloroplast exons directly (Medina et al., 2018; Heyduk et al., 2016a, 2016b).

The availability of public transcriptome data, including over 1400 green plants as part of the One Thousand Plants project (OneKP; Matasci et al., 2014), has simplified the process of probe design for many plant groups. Transcriptome sequence data are now available for most angiosperm plant families and can be used for Hyb‐Seq probe design. Predicted protein sequences from several species can be sorted into low‐copy orthogroups based on sequence similarity using tools such as OrthoFinder (Emms and Kelly, 2015). One disadvantage of the transcriptome‐only approach to probe design is that probes spanning intron boundaries will not be effective during sequence capture. Identification of intron‐exon boundaries is possible using MarkerMiner (Chamala et al., 2015), which aligns transcriptome data to reference genome sequences and returns intron‐masked multiple‐sequence alignments. If no reference genome is available, a low‐coverage genome sequence (10–15× coverage) can also be used to design probes around intron boundaries (Gardner et al., 2016). Finally, the pipeline Sondovač (Schmickl et al., 2016) uses a combination of transcriptome and genome skimming data to identify possible nuclear exons, and their introns, to be captured. There are many filtering steps in the pipeline to assure that the target loci are orthologous and putatively single copy.

It is generally advisable to design target enrichment probes using orthologous sequences from multiple species. In addition to ensuring the loci are truly single‐copy in the target taxa, probes designed from orthologous sequences will extend the breadth of phylogenetic utility of the probe set (Johnson et al., 2016; Villaverde et al., unpublished manuscript). Further discussion of bait design considerations can be found at: https://github.com/mossmatters/KewHybSeqWorkshop. A number of broad‐scale target enrichment projects are under development in plants, including by the Plant and Fungal Tree of Life (PAFTOL; Royal Botanical Gardens Kew, Richmond, Surrey, United Kingdom), Genealogy of Flagellate Plants (GoFlag; University of Florida, Gainesville, Florida, USA), and Anchored Phylogenomics (Léveillé‐Bourret et al., 2018) groups. The possibility of a universal set of genes that can be used for any plant species is an exciting future direction for targeted sequencing.

Being able to use herbarium specimens in phylogenomics analyses is one of the major advantages of the target enrichment approach. DNA from herbarium specimens is often degraded into very small fragments, meaning PCR‐based approaches are unsuccessful at amplifying loci. In contrast, target enrichment has proven successful for antique DNA collections in many organisms, including 100‐year‐old herbarium specimens (Villaverde et al., unpublished manuscript). Target enrichment also has an advantage over phylotranscriptomic methods in groups in which live tissue is difficult to collect but herbarium collections exist.

### Workflow easily accomplished in a modern molecular lab

A typical molecular lab workflow would involve six steps: DNA extraction, genomic DNA fragmentation via sonication, HTS library preparation, sequence capture, PCR, and sequencing. The sonication step may be omitted for many herbarium specimens, which typically have highly fragmented DNA. Depending on the methods of DNA extraction and library preparation, a 96‐well plate of samples may be prepared for sequencing in as little as two or three weeks. One difference between genome skimming and target enrichment is that it may not be economical to send DNA extracts to a third‐party for library preparation. The libraries would need to be returned to researchers for hybrid enrichment and then sent back for sequencing. Additional cost‐cutting measures may be used, including the preparation of streptavidin beads. For an example of one possible low‐cost workflow, see: https://github.com/mossmatters/KewHybSeqWorkshop.

If probe design is conducted using existing transcriptome and genomic resources, there is little up‐front cost for target enrichment studies. The typical cost for probe sequences and target enrichment reagents is US$200 per reaction with myBaits kits (Arbor Biosciences, Ann Arbor, Michigan, USA), and a single reaction can be used to enrich up to 96 Illumina libraries for hundreds of loci. The cost of library preparation is similar to other methods and can utilize typical DNA library preparation kits such as TruSeq Nano (Illumina Inc.) or more economical kits such as those provided by KAPA (Roche Sequencing, Pleasanton, California, USA) and NEBNext (New England BioLabs, Ipswich, Massachusetts, USA). Studies may employ different strategies for sequencing: for example, if capture of flanking intron regions is important, MiSeq 2 × 300 PE reads would be ideal, whereas if many herbarium specimens are used, a shorter read length may be a more appropriate choice.

### Data analysis is amendable to type of enrichment

After sequencing, data analysis involves reconstructing the loci from sequencing reads before proceeding to sequence alignment and phylogenetic reconstruction. Several pipelines have been developed to assist: for example, HybPiper (Johnson et al., 2016) was designed for Hyb‐Seq and exon‐based approaches, whereas Phyluce (Faircloth, 2015) was designed for the UCE approach. Users should pay special attention to the detection of paralogous sequences recovered by sequencing. In some cases, paralogous sequences may not affect further analysis (if they are recent enough to be monophyletic for each sample). In other cases, paralogs may prove useful to identify further loci for phylogenetics; when the relative age of a genome duplication is known, reads from the two paralogs can be sorted and assembled into separate, orthologous alignments (e.g., see Johnson et al., 2016).

Sequence alignments generated by target capture can be concatenated into a supermatrix or used for gene‐tree‐based methods of phylogenetic reconstruction, including ASTRAL‐III (Mirarab et al., 2014; Zhang et al., 2017), ASTRID (Vachaspati and Warnow, 2015), and BUCKy (Larget et al., 2010). This is especially useful with exon capture and Hyb‐Seq approaches, because loci are likely to be long enough to contain many variable sites and produce gene trees with high confidence (Folk et al., 2015; Johnson et al., 2016; Villaverde et al., unpublished manuscript). Filtering potential target genes to ensure the presence of long coding regions (>1000 bp) and, when intron position is known, long exon regions (>500 bp) will increase the probability that gene trees may be resolved. Recovery of organellar DNA as a byproduct of nuclear target enrichment can depend on many factors, including how much of the extraction contains organellar DNA and the efficiency of target enrichment. One method for increasing the off‐target organellar coverage is to add a dilution of the unenriched library to the post‐hybridization library (K. Weitemier, pers. comm.).

### Large segment enrichment

Recent advances in sequence capture have introduced the capacity to enrich for not only exons but also intergenic regions. One such method, known as region‐specific extraction (Dapprich et al., 2016), utilizes primers, designed in similar fashion as target enrichment probes, and a second‐strand synthesis using biotinylated nucleotides that facilitates the enrichment of long pieces of DNA using a standard magnet approach. This method allows for sampling of long and phylogenetically informative regions outside of exonic regions and has the potential to allow for easy assembly and identification of paralogs, acquisition of conserved regulatory regions, and identification of structural variation that would otherwise not be obtainable in taxa without fully sequenced genomes. Another approach is the use of capture probes on gene‐seized DNA fragments, followed by sequencing with long‐read technology such as Nanopore and PacBio (Giolai et al., 2016, 2017). This approach has the benefit of being very similar to general target enrichment, making adoption an easier transition. Another strength of these approaches is the possibility of targeted sequencing of large genomic regions in non‐model species, a powerful tool for both phylogenomics and evolutionary genomics.

## TRANSCRIPTOMES

Live tissue → RNA extraction, library preparation, and sequencing → transcriptome assembly → homology and orthology inference → phylogenetic inference

Using transcriptome sequencing to generate protein coding sequences for phylogenomics, or “phylotranscriptomics,” has the versatility to inform relationships from closely related species (Pease et al., 2016) to ancient relationships with relatively slow‐evolving coding sequences (Wickett et al., 2014). Transcriptomes contain rich information on both gene sequences and gene expression. No prior knowledge of sequences is required, and the transcriptome data generated for one project are reusable for a different project. Although phylotranscriptomics has been gaining popularity during the past several years, its use is still restricted to a relatively small number of research groups due to the relatively high cost per sample (US$260–350 plus labor) and logistics in obtaining and handling tissue due to unstable RNA molecules. In addition, data analysis of transcriptome data requires command line tools and overcoming computational challenges such as handling isoforms, incomplete/missing gene sequences, and misassembly. However, most of these hurdles have been lowered by recently developed equipment, commercially available kits, and analytical tools.

### Proper planning for collection of living tissue

Living collections, seed banks, and reputable commercial seed and live plant providers are the primary sources for tissue used for phylotranscriptomics. To supplement existing collections, there are two approaches that can be used to collect tissue samples suitable for transcriptome analysis from wild populations. RNA*later* (Thermo Fisher Scientific, Waltham, Massachusetts, USA) stabilizes both DNA and RNA and allows for collecting samples in ambient temperature. The key step for using RNA*later* is to slice thick tissue thin so that the solution penetrates tissue quickly. An alternative strategy, using liquid nitrogen, is logistically more challenging but enables a wider range of analyses in addition to DNA and RNA, such as protein and secondary metabolites (Sedio et al., 2018). Having collaborators based in a local institution near the field site allows for support in shipping and storing equipment and samples, especially if collecting internationally. Permits for collecting tissue for RNA isolation may be more difficult to obtain compared to those needed for silica‐preserved samples. Advanced planning and communication are essential for planning these trips. Once tissue samples are obtained, long‐term storage of either RNA*later*‐preserved or flash‐frozen materials can be expensive as they occupy freezer space and do not tolerate thawing. See Yang et al. (2017) for an example of a field collection and tissue storage workflow.

### Standardization of tissue may not be critical

Due to the dynamic nature of gene expression, one of the most frequently asked questions in phylotranscriptomic project design is what tissue to use. Traditionally, most transcriptome studies have focused on differentially expressed genes. For phylotranscriptomic purposes, we typically aim to recover as many genes as possible, especially housekeeping genes. Ideally a mixture of plant tissues should be used. However, logistic constraints of field collection often limit collection to vegetative tissues that vary in growth stages. Conditions such as temperature, day length, and time of day for collecting can be difficult to standardize but are less important when the goal is to recover housekeeping genes. A useful rule of thumb is to collect young leaves, flower buds, and meristems, which have a relatively high RNA concentration and are low in secondary metabolites compared to mature leaves, making them easier to work with (Johnson et al., 2012).

### Avoiding contamination in RNA extraction

A number of phylotranscriptomic protocols have been developed in various plant lineages (Johnson et al., 2012; Yockteng et al., 2013; Jordon‐Thaden et al., 2015; Yang et al., 2017). Suitable extraction protocols can be lineage specific, and we recommend starting from existing protocols that have proven effective in closely related plant groups. Despite having to keep tissue frozen until extraction, the RNA extraction procedure is quite similar to DNA extraction. RNA extraction is best done in small batches of 12 or less because it is necessary to move relatively quickly to avoid RNA degradation.

Extreme care should be taken to avoid contamination, especially from closely related plants. This is because sequencing coverage varies by several orders of magnitude among genes in any given transcriptome. Unlike DNA analysis, in which contamination can be filtered out by low sequence coverage, highly expressed genes from contaminants can be difficult to remove analytically, especially if they are from a closely related species. Unfortunately, publicly available transcriptome data retrieved from the National Center for Biotechnology Information (NCBI) Short Read Archive (SRA) often contain contaminated reads, or even hybrid or mixed samples. As *rbcL* and *matK* genes can often be recovered from transcriptomes, they can be used to compare against sequences in GenBank to detect potential contamination. The recently developed tool CroCo (Simion et al., 2018) can be used to detect potential cross‐contamination from transcriptome data sets generated by the same research group.

### Data analysis requires powerful computers and command line tools

Due to memory requirements for de novo transcriptome assembly, a high‐end desktop computer with at least 64 Gb of memory or high‐performance computing clusters are needed for data processing. Although point‐and‐click software platforms such as Galaxy (Afgan et al., 2016), CLC Genomics Workbench (QIAGEN, Valencia, California, USA), and DNA Subway (https://dnasubway.cyverse.org/) are available for de novo assembly, downstream analysis steps such as data filtering, homology and orthology inference, matrix construction, and gene tree analyses are still active areas of research and development. No existing point‐and‐click tools can properly handle the entire phylotranscriptomic workflow, and command line skills are required to properly analyze transcriptome data. We highly recommend familiarizing yourself with a scripting language (such as Python) and Unix command line tools through bioinformatics courses, workshops, and online classes (such as those offered through Coursera [https://www.coursera.org/]). Recently, Carey and Papin (2018) published a guide for biologists learning to program, which provides a practical resource for those just getting started. The “simple fool's guide” (De Wit et al., 2012) and the Eel Pond mRNAseq Protocol (http://khmer-protocols.readthedocs.io/en/v0.8.4/mrnaseq/index.html) are good examples to start with for data analysis, although updated protocols should be considered when available.

### Isoforms, incomplete sequences, and gene and genome duplication

With proper orthology inference and filtering, phylotranscriptomic data sets using housekeeping genes can achieve matrix occupancy similar to Sanger‐based methods (Yang and Smith, 2014; Yang et al., 2015, 2018). Methods developed without explicit consideration for gene and genome duplication events, such as HaMStR (Ebersberger et al., 2009) and OrthoMCL (Li et al., 2003), are potentially problematic in plants, especially when non‐single‐copy gene families are of interest. Recently developed tools such as OrthoFinder (Emms and Kelly, 2015), in our experience, perform better than OrthoMCL in retaining gene family structure instead of breaking gene families apart. Approaches such as constructing phylomes (the collection of gene phylogenies for a taxon; Huerta‐Cepas et al., 2011) followed by tree‐based orthology pruning (Yang et al., 2018) or all‐by‐all BLAST followed by Markov clustering and tree‐based orthology pruning (Yang and Smith, 2014) are more appropriate for the challenges of plant orthology inference, especially with complex gene and genome duplication scenarios. Optimal homology and orthology inference in plants is still an active research area, with novel tools being developed by multiple research groups including the Joint Genome Institute (https://phytozome.jgi.doe.gov). Multiple challenges remain in orthology inference: all‐by‐all homology search is computationally intensive (less of a concern with DIAMOND; Buchfink et al., 2015), inflation values have an unknown impact in Markov clustering (van Dongen, 2000), and *E*‐value saturation effects from BLAST are unknown. On the other hand, baited methods, such as building phylomes or sorting transcriptomes using a core set of orthogroups (e.g., McKain et al., 2016b), rely on a high‐quality core gene set or focal proteome, as errors and incompleteness in these sets can propagate into subsequent analyses.

### Detecting gene and genome duplication events

Due to the complexity of de novo transcriptome assembly, it is difficult to distinguish isoforms and alleles from recently duplicated paralogs. Our experience is that detecting polyploids formed during the past few million years is often difficult using transcriptomes due to paralog divergence, taxon sampling, and incomplete lineage sorting. Transcriptome data are suitable for detecting more ancient polyploidy events given proper homology inference (Li et al., 2015; McKain et al., 2016b; Yang et al., 2018). Large data sets, such as OneKP, have demonstrated the utility of such an approach through the identification of hundreds of whole genome duplication events (M. Barker, pers. comm.). Moving forward, some exciting aspects of phylotranscriptomic analysis include gene loss/silencing, relative expression levels, and substitution rates between paralogous pairs.

In summary, while sample handling is delicate, with transcriptome data, housekeeping genes can be used for species tree reconstruction, while gene duplication and loss/silencing can be used for functional inference. The learning curve for data analysis is steep, but the return allows for novel biological insights in non‐model systems.

## DISCUSSION

With so many phylogenomic methods available for plants, many limitations that previously plagued systematics projects can be alleviated through a multifaceted approach. Good planning with insight into the biology of one's system as well as into the potential limitations of the methods used can improve the likelihood of success. When planning a phylogenomic project, one should first consider data already available from public databases such as NCBI (GenBank, SRA, and the Transcriptome Shotgun Assembly Sequence Database [TSA]) and Phytozome (https://phytozome.jgi.doe.gov). It is increasingly common to start by summarizing and/or re‐analyzing existing data when designing phylogenomic projects. Existing data can give a researcher a head start, circumventing the need to generate preliminary data when they are necessary for certain approaches (e.g., microfluidic PCR and target enrichment). Although the phylogenomic community has been moving toward increased transparency and data/code sharing, more often than not it is frustrating to re‐use published data sets. As such, researchers should be sure to contribute responsibly to the community by making data and analyses openly available and well‐annotated with metadata. Additionally, vouchers should be made for samples to link a physical plant specimen to generated data (Funk et al., 2017). Here, we make recommendations on metadata and data sharing to optimize re‐usability of data and transparency of research (Table 2).

**Table 2:** Recommendations for data sharing and archiving

Archive format/platform	Best practice	Information to include	Special considerations
Vouchers	Deposit specimen with appropriate characteristics to identify to species level in a herbarium	GPS point, locality data, collector, collection number	Permits often required. Special permission for living collections (e.g., botanical gardens, arboreta)
NCBI Short Read Archive	Submit all raw read data	Taxon (as specific as possible); voucher information; tissue type; methods for collection, extraction, and library preparation; read type (paired or single end)	Submit biological replicates separately if sampling for RNA‐Seq experiment. Link populations and accessions together in a BioProject
Dryad	Provide details to reproduce results including commands, scripts, program versions, and log files. Major steps in data analysis should be included. Provide final data sets from which major conclusions are drawn	Cleaned and assembled reads; intermediate and final analysis files; parameters for analyses; scripts as used in associated analyses; details not presented in manuscript but necessary to replicate results	Links to Github, Bitbucket, or other online repositories for updated versions of scripts; simply stating “custom scripts” is not acceptable. Provide documentation of code used and parameters, such as a Jupyter Notebook (http://jupyter.org), to promote repeatability

Given your short‐ and long‐term research goals, consider the longevity of the data generated and ask the question: would my data become obsolete in five years? Tissue collection should take into consideration the improvement of phylogenomic approaches, as well as other future approaches. For example, is collecting seeds and frozen or RNA*later*‐preserved tissue in addition to silica‐preserved samples feasible for at least some species? These will provide resources for future transcriptome and whole genome sequencing, even if it is not the current goal of a project. With the advance of whole genome sequencing, a well‐curated living collection will become increasingly important not only to you but to the community.

Multiple approaches may be combined for developing phylogenomic projects. Transcriptomes and genomes can be used to design Hyb‐Seq and PCR primers. Whole genomes can be used for reference‐based mapping of transcriptome, RAD‐seq, and target enrichment data and help inform lineages with recent divergence or phase homologous sequences. Combinations of approaches can provide novel insight into relationships by using the strengths of these approaches to inform each other. For example, identification of a hybridization event is possible through most of the approaches depicted here. The uniparental inheritance of organellar genomes recovered from genome skimming can elucidate the parental history of a hybrid, identifying the maternal genome donor and determining whether the event is unidirectional or bidirectional.

Finally, it is becoming increasingly attractive to develop “model clades” with a combination of whole genomes, transcriptomes, Hyb‐Seq/genome skimming at species level, and RAD‐seq at population level. With a suite of tools, we can not only reconstruct the evolutionary history of these clades but also start asking questions about genetic mechanisms underlying trait evolution and adaptation. Phylogenomic methods provide much more than just evolutionary history, they provide insight into different aspects of a plant's genomes, which can lead to novel discoveries in previously intractable lineages.
